# A Model for Determining Strength for Embedded Elliptical Crack in Ultra-high-temperature Ceramics

**DOI:** 10.3390/ma8085018

**Published:** 2015-08-05

**Authors:** Ruzhuan Wang, Weiguo Li

**Affiliations:** 1 Chongqing Key Laboratory of Heterogeneous Material Mechanics, College of Aerospace Engineering, Chongqing University, Chongqing 400030, China; E-Mail: wrz@cqu.edu.cn; 2 State Key Lab for Strength and Vibration of Mechanical Structures, Xi’an Jiaotong University, Xi’an 710049, China

**Keywords:** embedded elliptical crack, ultra-high-temperature ceramics, fracture strength, theoretical model, ABAQUS

## Abstract

A fracture strength model applied at room temperature for embedded elliptical crack in brittle solid was obtained. With further research on the effects of various physical mechanisms on material strength, a thermo-damage strength model for ultra-high-temperature ceramics was applied to each temperature phase. Fracture strength of TiC and the changing trends with elliptical crack shape variations under different temperatures were studied. The study showed that under low temperature, the strength is sensitive to the crack shape variation; as the temperature increases, the sensitivities become smaller. The size of ellipse’s minor axes has great effect on the material strength when the ratio of ellipse’s minor and major axes is lower than 0.5, even under relatively high temperatures. The effect of the minor axes of added particle on material properties thus should be considered under this condition. As the crack area is set, the fracture strength decreases firstly and then increases with the increase of ratio of ellipse’s minor and major axes, and the turning point is 0.5. It suggests that for the added particles the ratio of ellipse’s minor and major axes should not be 0.5. All conclusions significantly coincided with the results obtained by using the finite element software ABAQUS.

## 1. Introduction

Ultra-high temperature ceramics (UHTCs) such as the transition metal borides and carbides have melting points higher than 3000 °C, which can be used in the high temperature and oxidizing environments and have good chemical and physical stabilities. These materials have been developed for the leading edge and nose cap materials in hypersonic vehicles. The addition of SiC particles or sheets has been proved to be the most promising way for the improvement of the oxidation resistance and mechanical performances of UHTCs [1,2,3,4]. The SiC grains in the microstructure of UHTCs are nearly to be the ellipse [3]. Considering that the UHTCs used in high-temperature applications, it is very important and necessary to study their mechanical performances at high temperatures. At high temperatures the SiC will oxidize, which thus can lead to the formation of elliptical crack in the microstructures. These formed cracks would affect the material strength. Also, the SiC inclusions have been identified as the critical flaw in dense, fine grained materials, and the size of major axes of elliptical particle is considered to be the flaw size of materials [3]. Therefore, the effects of elliptical flaw on the mechanical properties of UHTCs under different temperatures should be taken into account. In this current paper, we assume the flaw as the embedded elliptical crack. We will study the control mechanisms of material strength of UHTCs including the embedded elliptical crack under different temperatures.

The study on embedded crack, particularly on embedded elliptical crack, has always been important in linear elastic fracture mechanics. In recent decades, mechanical workers have attempted to develop applicable theories for embedded elliptical crack in brittle solid, initially establishing the widely accepted theoretical frameworks under normal or slightly higher temperatures. Green and Sneddon [5] analyzed the stress and strain fields on the embedded elliptical crack surface. The elastic matrix was used under a far field uniaxial tensile stress, and the opening displacement of the crack surface was obtained. Irwin [6] proposed the commonly used stress intensity factor model for embedded elliptical crack using the elastic matrix subjected to uniform tensile stress. Shah and Kobayashi [7] obtained the stress intensity factor at the tip of elliptical crack under arbitrary normal loading. Chen *et al.* [8] evaluated the stress intensity factor of elliptical crack by using differential-integral equations. Atroshchenko *et al.* [9] proposed the stress intensity factor for any elliptical crack embedded in a homogeneous elastic medium using the weight function concept.

However, the above methods do not consider the effect of temperature. Mechanical performances and fracture mechanisms under high temperatures differ significantly from those under low temperatures. Thus, many fracture theories are not applicable at high temperatures. Although several such fracture theories for high temperatures have achieved some progress [10,11,12], fracture theories of embedded elliptical crack considering the effect of temperature are almost non-existent. Moreover, as the experiment is difficult to simulate that at high temperatures, the theoretical research is necessary.

In this work, a fracture strength theoretical model was applied to an embedded elliptical crack in brittle solid at room temperature. The model was based on the stress intensity factor model proposed by Irwin and the fracture criterion proposed by Griffith. Through further research on effects of various physical mechanisms on material strength, a thermo-damage strength theoretical model for UHTCs was established and applied to each temperature phase. Fracture strength of TiC and the changing trends with the elliptical crack shape variations under different temperatures were studied and then compared with the results obtained by using the finite element software ABAQUS (ABAQUS, Inc., Pawtucket, RI, USA).

## 2. Thermo-Damage Strength Theoretical Model

**Figure 1:**
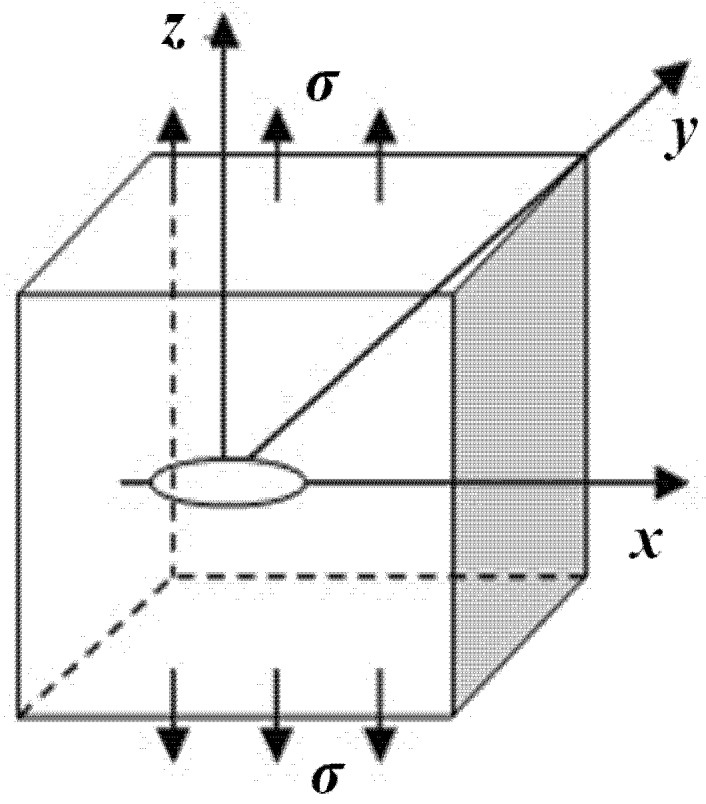
Infinite elastic matrix with an embedded elliptical crack subjected to uniform tensile stress.

**Figure 2:**
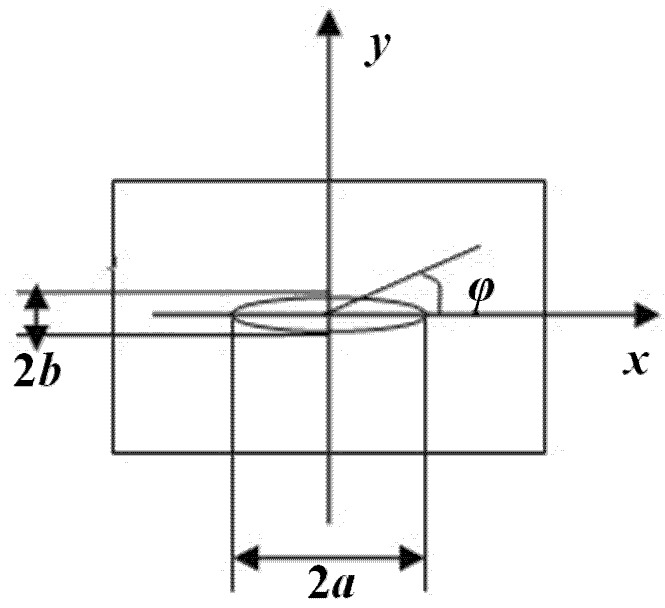
Floor plan of the embedded elliptical crack.

The following is the stress intensity factor model for an embedded elliptical crack in the elastic matrix subjected to uniform tensile stress as proposed by Irwin [6]:

(1) KI=σπE(k)(ba)12(a2sin2φ+b2cos2φ)14

(2) E(k)=∫0π2(1−k2sin2α)12dα,k2=a2−b2a2

where *K*_I_ is the stress intensity factor of an embedded elliptical crack; *E*(*k*) is an elliptical integral of the second kind; σ is the uniform tensile stress; *a* and *b* are one-half of the ellipse’s major and minor axes, respectively; φ is the polar angle from the ellipse center; α is the integral angle; *k* is a function of *a* and *b*. Floor plans of the embedded elliptical crack are as shown in Figure 1 and Figure 2.

The Griffith fracture criterion states that the fracture of brittle materials initiates at the crack edge with the largest stress intensity factor. When this largest stress intensity factor is equal to the fracture toughness of materials, the fracture of materials occurs. In this work, the stress intensity factor is predicted by using Equation (1), and the largest stress intensity factor *K*_max_ is removed. Then, letting Kmax=KIC=[2γE01−ν2]12,σ=σ0f, the fracture strength model under room temperature is obtained as follows:

(3) σ0f=(2aγE0πb(1−ν2))1/2(a2sin2φm+b2cos2φm)−14E(k)

where *K*_IC_ is the fracture toughness of materials; $\sigma_{\text{0}}^{\text{f}}$ is the fracture strength under room temperature; *E*_0_ is the Young’s modulus; ν is the Poisson’s ratio; γ is the fracture surface energy; φ_m_ is the polar angle of the point where the stress intensity factor is largest from the ellipse center.

In our previous work [10], based on the following assumptions: “(1) There is maximum energy storage for a particular material. This energy can be supplied by both strain energy and heat energy; (2) There is a quantity equivalent relation between strain energy and heat energy”, a temperature dependent fracture strength model for the UHTCs with a reference temperature of 0 °C was obtained as follows:

(4) $$\sigma_{\text{th}}\left(T\right)={\left[\frac{{\left(\sigma_{\text{th}}^{\text{0}}\right)}^{2}}{E_{\text{0}}}\cdot E\left(T\right)\left[\text{1}-\frac{1}{\int_{0}^{T_{\text{m}}}C_{\text{p}}\left(T\right)dT+\Delta H_{\text{M}}}\int_{0}^{T}C_{\text{p}}\left(T\right)dT\right]\right]}^{1/2}$$

where σ_th_(*T*) is the temperature dependent fracture strength of materials; $\sigma_{\text{th}}^{\text{0}}$ is the fracture strength at the same initial damage state and reference temperature; *E*(*T*) is the temperature dependent Young’s modulus; *C*_p_(*T*) is the specific heat capacity for constant p pressure and temperature *T*; *T*_m_ is the melting point; ∆*H*_M_ is the latent heat of melting.

As is well known, fracture is a very complex process that involves the combined effects of temperature and microstructures. There has been no one set theory “set in stone” to handle all of the factors in fracture, especially at high temperatures. The studies have shown that under low temperatures the fracture of brittle materials is sensitive to the single crack size, while as the temperature increases the sensitivity decreases. It can be observed that for the fracture mechanisms of UHTCs under different temperatures the temperature and damage should be first concerned problems. Based on the above temperature dependent fracture strength model, we developed a simple method to consider both the effects of temperature and damage on the material strength, which can be expressed as follows [11]:

(5) $$\sigma_{\text{th}}(T,l)=\sigma_{\text{th}}\left(T\right)\sigma_{\text{0}}^{\text{f}}\left(l\right)/\sigma_{\text{th}}^{\text{0}}$$

Where $\sigma_{\text{th}}\text{(}T,l\text{)}$ is the temperature-damage-dependent fracture strength; $\sigma_{\text{0}}^{\text{f}}\left(l\right)$ is the damage-dependent fracture strength with respect to some reference temperature; the term $\sigma_{\text{0}}^{\text{f}}\left(l\right)/\sigma_{\text{th}}^{\text{0}}$ describes the effects of damage term on the fracture strength; *l* corresponds to the damage term.

Similarly, we now proceed to develop the fracture strength model considering the effects of temperature and the embedded elliptical crack, which can be obtained as follows:

(6) $$\sigma_{\text{th}}(T,a,b,\varphi_{\text{m}})=\sigma_{\text{th}}\left(T\right)\sigma_{\text{0}}^{\text{f}}/\sigma_{\text{th}}^{\text{0}}$$

where $\sigma_{\text{th}}(T,a,b,\varphi_{\text{m}})$ is the fracture strength model considering effects of temperature and the shape parameters of the embedded elliptical crack.

Based on the above analyses, the fracture strength model can be formulated:

(7) σth(T,a,b,φm)=σth(T)σ0f/σth0=[2aγE(T)πb(1−ν2)[1−1∫0TmCp(T)dT+ΔHM∫0TCp(T)dT]]1/2(a2sin2φm+b2cos2φm)−14E(k)

The model (Equation (7)) establishes a simple quantitative relationship between the fracture strength of materials, temperature, elliptical crack shape variations and basic material parameters which can be obtained easily through experimental method and material handbook.

## 3. Results and Discussion

Table 1 shows the relative parameters [13,14,15,16] obtained from experiments. Using the above thermo-damage strength theoretical model (Equation (7)), the fracture strength of TiC and the changing trends with the elliptical crack shape variations under different temperatures were studied in detail.

**Table 1:** Material properties of TiC [13,14,15,16].

Material Parameters	Values and Expressions
*E*_0_ (GPa)	444
*E*(*T*) (GPa)	See the reference [13]
ν	0.195
γ (J·m^−^^2^)	15.808
*T*_m_ (°C)	3056.85
∆*H*_M_ (cal/mol)	17000
*C*_p_(*T*) (cal/mol)	11.94 + 0.23 × 10^−^^3^*T* − 3.53 × 10^5^*T*^−^^2^ + 0.45 × 10^−^^6^*T*^2^

**Figure 3:**
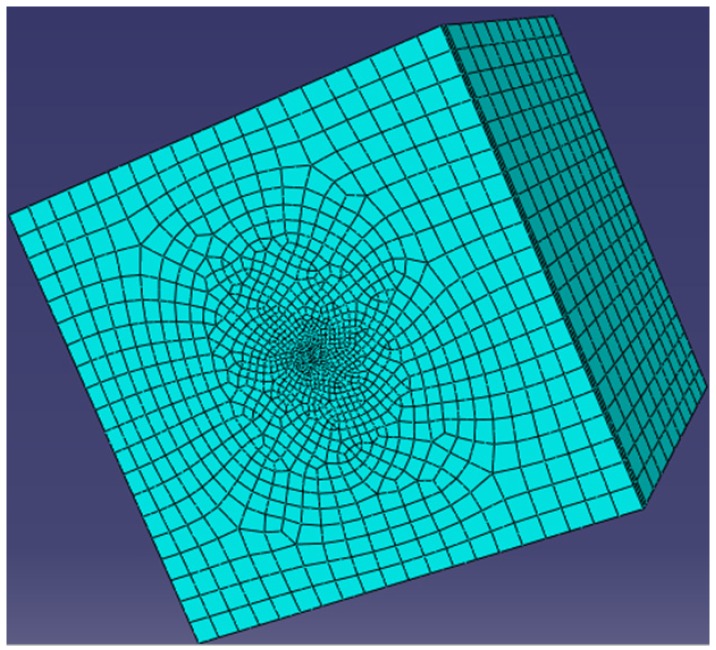
Finite element mesh.

In this work, the finite element software ABAQUS was used to verify the above trends. We consider the model to be an infinite elastic matrix with an embedded elliptical crack. According to the Saint-Venant’s principle, the ratio of the largest half of the ellipse’s major axis and the side of the matrix paralleling to the major axis was set to 1:20. The geometry sizes were set to 400 × 400 × 400 (thickness × width× length). The *z* indirection constraints were imposed on the matrix surface where the elliptical crack was located, and the uniform tensile load was applied to its opposite surface. The finite element mesh is as shown in Figure 3. The unit type used the second-order, reduced-integration hexahedral elements (C3D20R). The material parameters used were temperature dependent. The stress intensity factor near the crack tip, according to the fracture mechanics theory, is as follows:

(8) $$K_{\text{I}}=\underset{r\to 0}{\lim}\sqrt{2\pi r}\sigma (x,y,0)$$

where *r* is the geometric parameter characterization of the size of crack tip, and *r* << *a*, *r* << *b*. Here, *r* is set to be a very small value, and the stress intensity factor thus depends on σ (*x*, *y*, 0). The biggest stresses σ (*x*, *y*, 0) on the crack edge under different temperatures were calculated by ABAQUS. Using the biggest stress σ (*x*, *y*, 0), the trend of the largest stress intensity factor on the crack edge with *b*/*a* ratio changes can be determined. Then the trend of the fracture strength with changes in *b*/*a* ratio can be obtained. This is because the trend of the fracture strength with crack shape variation is just completely opposite to the trend of the biggest stress intensity factor with crack shape variation.

**Figure 4:**
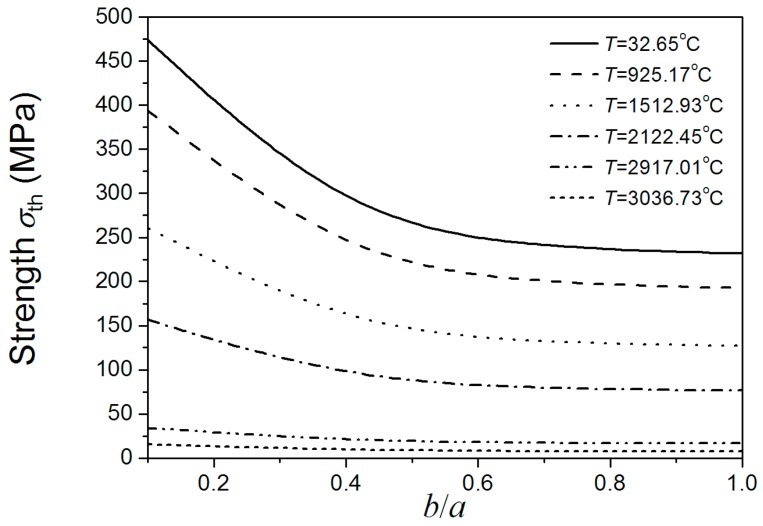
*b*/*a* ratio dependence of the material’s fracture strength under different temperatures (*a* is a definite value).

**Figure 5:**
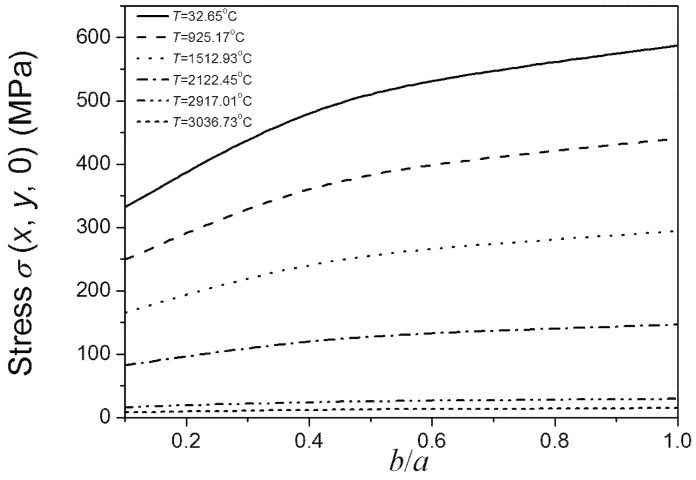
*b*/*a* ratio dependence of the highest σ (*x*, *y*, 0) on the crack edge under different temperatures (*a* is a definite value).

Figure 4 shows that the material strength decreases as the size of *b* increases. The size of *b* has great effect on the material strength when the *b*/*a* ratio is lower than 0.5, even under relatively high temperatures. While the current research usually did not consider the effect of the minor axes of added particle on material properties, which just considered the effect of major axes [3]. If under this condition, the effect of the minor axes of added particle should be taken into account. From the viewpoint of the maintenance of high fracture strength of materials under different temperatures, if the major axes of added elliptical particle is a certain value, the ratio of minor axes and major axes of added elliptical particle should be lower than 0.5. When the *b*/*a* ratio is higher than 0.5, the change of *b* has little effect on the material strength, even under low temperatures. Under this condition, the effect of the minor axes of added particle can be negligible. It also can be seen form Figure 4 that under low temperatures, strength is sensitive to the size of *b* when *b*/*a* ratio is lower than 0.5. As the temperature increases, the sensitivity of material strength to the size of *b* decreases. Under super high temperatures, the effects of the size of *b* on material strength compared to temperature are negligible. Comparing Figure 4 and Figure 5, the above conclusions obtained by Equation (7) coincide with the results obtained by using the finite element software ABAQUS. This indicates the rationality and applicability of our proposed method considering both effects of temperature and damage on fracture of materials.

**Figure 6:**
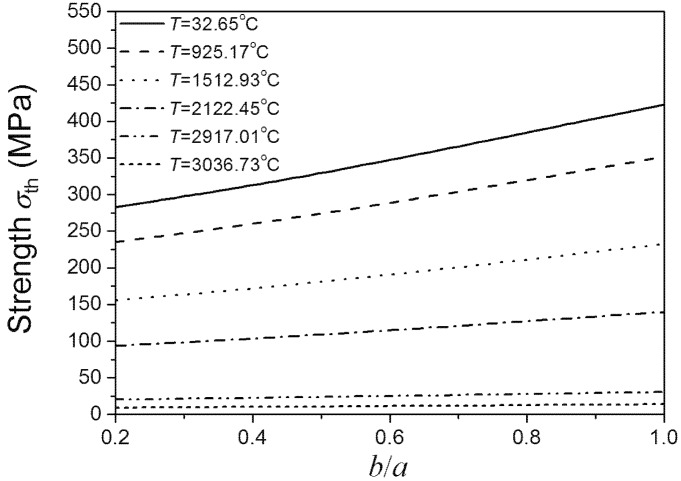
*b*/*a* ratio dependence of the material’s fracture strength under different temperatures (*b* is a definite value).

**Figure 7:**
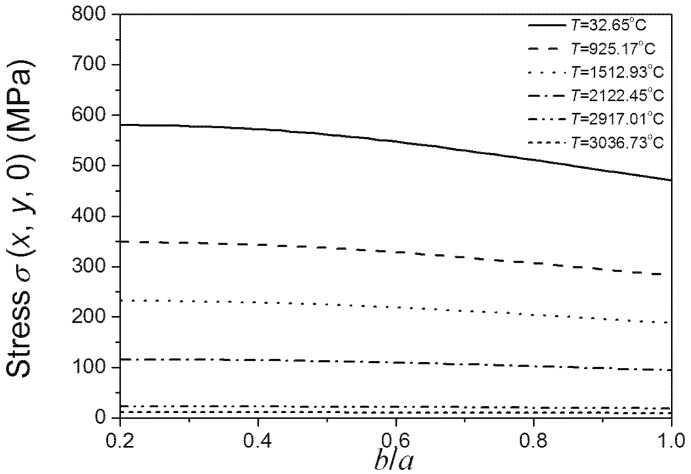
*b*/*a* ratio dependence of the highest σ (*x*, *y*, 0) on the crack edge under different temperatures (*b* is a definite value).

Figure 6 shows that material strength decreases as the size of *a* increases. The relationship between the fracture strength of materials and the size of *a* is nearly linear. It can be observed that whatever the value of *b*/*a* is the size of *a* is always the main control mechanism of material strength; even under the relatively high temperatures (about 2000 °C). It suggests that when preparing the UHTCs reducing the major axes of added elliptical particle should be firstly considered. This coincides with the experimental results reported by Watts *et al.* [3]. Moreover; we consider that improving the sintering method such as increasing the heating rate during hot pressing would result in the great strength of materials having small major axes of added elliptical grain. As can be seen from Figure 6; under low temperature; the fracture strength of materials is sensitive to the size of *a*. While under high temperatures; the sensitivity is little; and the effects of the size of *a* on fracture strength of materials compared to temperature are negligible. Comparing Figure 6 and Figure 7; the above conclusions obtained by Equation (7) agree well with the results obtained by using the finite element software ABAQUS. This indicates the applicability of our proposed temperature-damage dependent fracture strength model again.

**Figure 8:**
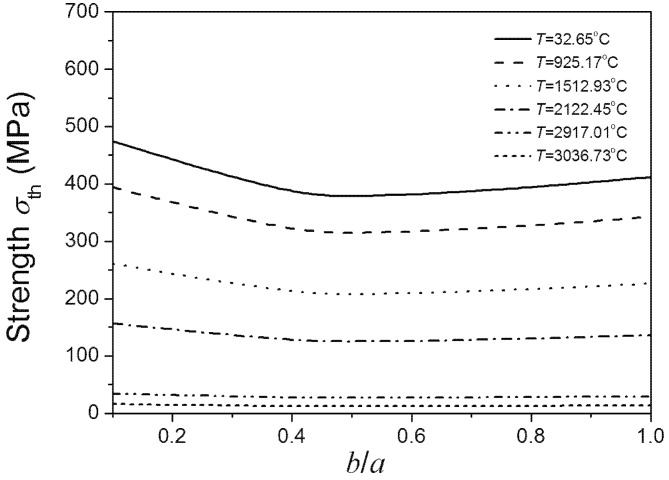
*b*/*a* ratio dependence of the material’s fracture strength on the same crack edge under different temperatures.

**Figure 9:**
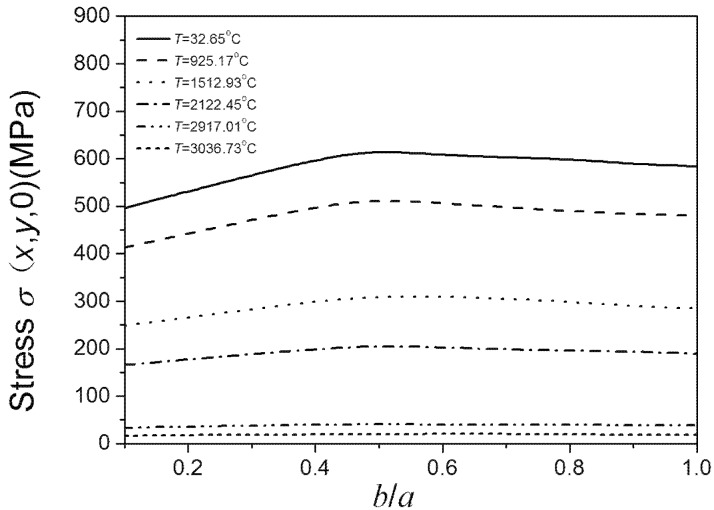
*b*/*a* ratio dependence of the highest σ(x,y,0) on the same crack edge under different temperatures.

Figure 8 shows the relationship between the temperature dependent fracture strength of materials and *b*/*a* ratio as the crack area is set. It can be observed that fracture strength decreases as temperature increases. The strength of material initially decreases with the increase of *b*/*a* ratio. The strength of material reaches its lowest point when the *b*/*a* ratio is 0.5, coinciding with the literature stating that on the same crack edge the stress intensity factor of the crack is largest when the *b*/*a* ratio is 0.5. However, when the *b*/*a* ratio increases from 0.5, the fracture strength starts increasing. Therefore, the fracture strength appears to have two maxima, when the *b*/*a* ratios are very small and equal to 1. It suggests that for the added particles the ratio of ellipse’s minor and major axes should not equal to 0.5. Figure 8 also shows that under low temperature, fracture strength is sensitive to *b*/*a* ratio lower than 0.5. However, when the *b*/*a* ratio is higher than 0.5, the trend of material strength with shape variation is more relaxed. The material strength is not sensitive to the *b*/*a* ratio under super high temperatures. Comparing Figure 8 and Figure 9, the above conclusions obtained by Equation (7) coincide with the results obtained by using the finite element software ABAQUS.

## 4. Conclusions

In this work, a fracture strength theoretical model applied to room temperature for embedded elliptical crack in brittle solid was obtained. The model is based on the stress intensity factor model for embedded elliptical crack proposed by Irwin and the fracture criterion proposed by Griffith. Further research on the effects of various physical mechanisms on material strength established a thermo-damage strength theoretical model for UHTCs applied to each temperature phase. This model was verified by comparison with results obtained by using the finite element software ABAQUS. The study showed that under low temperature, the strength is sensitive to the crack shape variation; as the temperature increases, the sensitivity decreases; under super high temperatures, the effects of crack shape on fracture strength of materials compared to temperature are negligible. As the size of *a* increases, the fracture strength of materials decreases lineally. When preparing the UHTCs reducing the major axes of added elliptical particle should be firstly considered. The size of *b* also has great effect on the material strength when the *b*/*a* ratio is lower than 0.5, even under relatively high temperatures. The effect of the minor axes of added particle on material properties thus should be considered under this condition. As the crack area is set, the fracture strength decreases firstly and then increases with the increase of *b*/*a* ratio, and the turning point is 0.5. It suggests that for the added particles the *b*/*a* ratio should not be equal to 0.5. This study will provide a theoretical basis and guidance on the design and preparation of the UHTCs.
